# Pathophysiological significance of the two-pore domain K^+^ channel K_2P_5.1 in splenic CD4^+^CD25^−^ T cell subset from a chemically-induced murine inflammatory bowel disease model

**DOI:** 10.3389/fphys.2015.00299

**Published:** 2015-10-27

**Authors:** Sawa Nakakura, Miki Matsui, Aya Sato, Mizuki Ishii, Kyoko Endo, Sayaka Muragishi, Miki Murase, Hiroaki Kito, Hiroki Niguma, Natsumi Kurokawa, Masanori Fujii, Masatake Araki, Kimi Araki, Susumu Ohya

**Affiliations:** ^1^Department of Pharmacology, Division of Pathological Sciences, Kyoto Pharmaceutical UniversityKyoto, Japan; ^2^Institute of Resource Development and Analysis, Kumamoto UniversityKumamoto, Japan

**Keywords:** background K^+^ channel, K_2P_5.1, CD4^+^ T cell, inflammatory bowel disease, Ca^2+^ influx, cytokine production

## Abstract

The alkaline pH-activated, two-pore domain K^+^ channel K_2P_5.1 (also known as TASK2/KCNK5) plays an important role in maintaining the resting membrane potential, and contributes to the control of Ca^2+^ signaling in several types of cells. Recent studies highlighted the potential role of the K_2P_5.1 K^+^ channel in the pathogenesis of autoimmune diseases such as rheumatoid arthritis and multiple sclerosis. The aim of the present study was to elucidate the pathological significance of the K_2P_5.1 K^+^ channel in inflammatory bowel disease (IBD). The degrees of colitis, colonic epithelial damage, and colonic inflammation were quantified in the dextran sulfate sodium-induced mouse IBD model by macroscopic and histological scoring systems. The expression and functional activity of K_2P_5.1 in splenic CD4^+^ T cells were measured using real-time PCR, Western blot, and fluorescence imaging assays. A significant increase was observed in the expression of K_2P_5.1 in the splenic CD4^+^ T cells of the IBD model. Concomitant with this increase, the hyperpolarization response induced by extracellular alkaline pH was significantly larger in the IBD model with the corresponding intracellular Ca^2+^ rises. The expression of K_2P_5.1 was higher in CD4^+^CD25^−^ T cells than in CD4^+^CD25^+^ regulatory T cells. The knockout of K_2P_5.1 in mice significantly suppressed the disease responses implicated in the IBD model. Alternations in intracellular Ca^2+^ signaling following the dysregulated expression of K_2P_5.1 were associated with the disease pathogenesis of IBD. The results of the present study suggest that the K_2P_5.1 K^+^ channel in CD4^+^CD25^−^ T cell subset is a potential therapeutic target and biomarker for IBD.

## Introduction

Due to the overlap in the pathogeneses and pharmacological treatments, Crohn's disease (CD) and ulcerative colitis (UC) are types of inflammatory bowel diseases (IBD) (Bouma and Strober, 2003; McCole, 2014). Dextran sulfate sodium (DSS) is known to chemically induce the pathogenesis of CD and UC in rodents (Okayasu et al., 1990; Dieleman et al., 1994). A genome-wide expression profiling study showed that DSS-associated genes in mice correlated with data obtained from IBD patients (Fang et al., 2011). Therefore, the DSS-induced IBD model is a commonly used model of IBD in mice, and a number of studies have used the DSS-induced IBD model to investigate the pathogenesis of CD and UC.

An elevation in intracellular Ca^2+^ by the release of Ca^2+^ from intracellular Ca^2+^ stores and Ca^2+^ influx through store-operated Ca^2+^ channels is one of the essential triggering signals for T cell activation (Cahalan and Chandy, 2009; Hogan et al., 2010). The K^+^ channel is also one of the key molecules that modulates Ca^2+^ signaling in T cells because it provides an electrochemical gradient to drive Ca^2+^ influx, and two major K^+^ channels (the voltage-gated, K_*V*_1.3 and intermediate-conductance Ca^2+^-activated, K_Ca_3.1) function in T and B lymphocytes (Cahalan and Chandy, 2009). Recent studies including our previous study demonstrated that the upregulation of ion channels (Orai/STIM, TRPV1, TRPA1, and K_Ca_3.1) in CD4^+^ T cells was involved in the pathogenesis of IBD, while their pharmacological blockade elicited a significant decrease in IBD disease severity (Di Sabatino et al., 2009; Di et al., 2010; Bertin et al., 2014; Kun et al., 2014; Ohya et al., 2014).

The background or leak two-pore domain K^+^ (K_2P_) channel superfamily includes 16 members and plays a crucial role in diverse physiological functions through the regulation of cell excitability (Enyedi and Czirják, 2010; Es-Salah-Lamoureux et al., 2010). The K_2P_5.1 K^+^ channel (also known as TASK2/KCNK5) is activated by extra- and intracellular alkalization, and plays an important role in maintaining the resting membrane potential and the control of Ca^2+^ signaling in various types of cells. K_2P_5.1 has been shown to control diverse physiological and pathophysiological processes including innate immunity and cancer progression (Bittner et al., 2010b; Clark et al., 2011; Cid et al., 2013), and is, therefore, expected to become a potential therapeutic target for the treatment of autoimmune, inflammatory, and allergic diseases as well as several types of cancers. Recent studies described the pathophysiological impact of the upregulation of K_2P_5.1 in CD4^+^ and CD8^+^ T cells on the pathogenesis of autoimmune diseases such as rheumatoid arthritis and multiple sclerosis (Bittner et al., 2010a, 2011). K_2P_5.1 is inhibited by non-selective K^+^ channel inhibitors such as quinidine, lidocaine, and clofilium and is activated by volatile anesthetics (Wulff and Zhorov, 2008); however, the lack of selective blockers is represents an important difficulty in experimental studies on K_2P_5.1.

In the present study, we examined the involvement of the K_2P_5.1 K^+^ channel in CD4^+^ T lymphocytes in the pathogenesis of IBD using a mouse model of chemically-induced IBD. We further compared histopathologies between the DSS-induced IBD model using wild-type and K_2P_5.1-deficient mice.

## Materials and methods

### Preparation of the dextran sulfate sodium (DSS)-induced mouse IBD model

Male C57BL/6J (6–7 weeks of age) mice were purchased from Shimizu Laboratory Supplies (Kyoto, Japan), and were acclimatized for 1 week before the experiment. They were given distilled water containing 5.0% (wt/vol) dextran sulfate sodium 5000 (DSS) (Wako Pure Chemical, Osaka, Japan) *ad libitum*. Control mice were given drinking water only. Seven days after the administration of DSS, mice were sacrificed, tissue samples were taken, and colitis and inflammation were assessed macroscopically and scored as previously reported (Ohya et al., 2014). All experiments were carried out in accordance with the guiding principles for the care and use of laboratory animals in Kyoto Pharmaceutical University, and the protocols were approved by the committee on the Ethics of Animal Research of Kyoto Pharmaceutical University (Permit Number: 15-12-091). K_2P_5.1 heterozygous knockout mice bred in a C57BL6 background [B6;CB-Kcnk5Gt(pU-21)81Imeg] were established by the exchangeable gene trap method (Araki et al., 2014). Homozygous (K_2P_5.1^−/−^) knockout mice were generated by crossing heterozygous (K_2P_5.1^+/−^) males with K_2P_5.1^+/−^ females (Cid et al., 2013). Genomic DNA was isolated from finger or tail biopsies following a 3-h digestion at 50°C in buffer containing 50 mM Tris-HCl (pH 8.0), 100 mM EDTA, 100 mM NaCl, 0.1% SDS, and 1 mg/mL proteinase K, followed by heat inactivation. PCR was performed using the primer pairs to distinguish the *K*_2P_*5.1* wild type (forward: 5′-GCTGAGAACAATAGGGACAG-3′ and reverse: 5′-TCACCCAGCTTTGGGATTCC-3′) and gene trapped (forward: 5′-GCTGAGAACAATAGGGACAG-3′and reverse: 5′-TACAGGCATCGTGGTGTCAC-3′). PCR conditions were as follows: 30 cycles at 94°C for 30 s, 60°C for 30 s, and 68°C for 30 s. The amplified products were separated on 1.0% agarose gels, and visualized by ethidium bromide staining. DSS-induced IBD model mice were prepared using male and female K_2P_5.1^−/−^ (6–10 weeks of age) and K_2P_5.1^+/−^ (6 weeks of age) mice.

### Histological scoring

Inflammation and crypt damage in hematoxylin and eosin (H&E)-stained sections were assessed as reported previously (Dieleman et al., 1994; Ohya et al., 2014). Briefly, for histological assessments, 1 cm of tissue collected from the distal colon was fixed in 10% buffered formalin, embedded in a paraffin block, cut into 5-μm-thick sections, and stained with H&E. The inflammation score was determined as a multiplication of the severity grade of inflammation (grade 0, none; grade 1, slight; grade 2, moderate; grade 3, severe) as well as its extent (grade 0, none; grade 1, mucosa; grade 2, mucosa, and submucosa; grade 3, transmural). The crypt damage score was determined as a multiplication of the damage grade of the crypt (grade 0, none; grade 1, basal 1/3 damage; grade 2, basal 2/3 damage; grade 3 only the surface epithelium intact; grade 4, entire crypt, and epithelium lost) and the percent area score (grade 1, 1–25%; grade 2, 25–50%; grade 3, 51–75%; grade 4, 76–100%). Data were obtained from three sections of the colon measured at least 200 μm apart per animal from four individual mice per group.

### Cell isolation, RNA extraction, reverse transcription, and real-time PCR

Total RNA was extracted and reverse transcribed from the T cells of mouse tissues (Ohya et al., 2013, 2014). Single cell suspensions were prepared by pressing the spleen (also the thymus and mesenteric lymph nodes) with a frosted grass slide and then filtering through a nylon mesh. CD4^+^ cells were isolated from cell suspensions by Dynabeads® FlowComp™ Mouse CD4^+^ (Invitrogen, Grand Island, NY, USA), according to the experimental manual supplied by Invitrogen, and were then collected in phosphate buffered saline (PBS) supplemented with 0.1% bovine serum albumin. A flow cytometric analysis confirmed that more than 95% of the purified T cells were CD4^+^. CD4^+^CD25^−^ and CD4^+^CD25^+^ cells were also isolated from cell suspensions by Dynabeads® FlowComp™ Mouse CD4^+^ CD25^+^ Treg Cells (Invitrogen). A flow cytometric analysis confirmed that more than 90% of purified T cells were CD4^+^CD25^−^ and CD4^+^CD25^+^. The resulting cDNA products were amplified with gene-specific primers, and primers were designated using Primer Express™ software (Ver 1.5, Applied Biosystems, Foster City, CA, USA). Quantitative, real time PCR was performed using Syber Green chemistry (SYBR® *Premix Ex Taq*™ II) (TaKaRa BIO, Osaka, Japan) on an ABI 7500 sequence detector system (Applied Biosystems) as previously reported (Ohya et al., 2014). The following PCR primers for mouse clones were used for real-time PCR: K_2P_5.1 (GenBank accession number: NM_021542, 722-844), amplicon = 123 bp; K_2P_3.1 (AF065162, 692-812), 121 bp; K_2P_9.1 (NM_001033876, 757-877), 121 bp; K_2P_16.1 (NM_029006, 191-312), 122 bp; interferon (IFN)-γ (NM_008337, 222-323), 102 bp; interleukin-4 (IL-4) (NM_021283, 126-246), 121 bp; IL-10 (NM_010548, 245-355), 111 bp; CD25 (NM_008367, 522-642), 121 bp; IL-17A (NM_010552, 165-277), 113 bp; β-actin (ACTB) (NM_031144, 419-519), 101 bp. Regression analyses of the mean values of three multiplex RT-PCRs for log_10_ diluted cDNA were used to generate standard curves (Ohya et al., 2014). Unknown quantities relative to the standard curve for a particular set of primers were calculated, yielding the transcriptional quantitation of gene products relative to the endogenous standard, ACTB. The following PCR primers were used for real-time PCR to confirm the knockout of K_2P_5.1 transcripts in T cells from K_2P_5.1^−/−^ mice: K_2P_5.1 (NM_021542, 472-567), 96 bp.

### Western blotting

Splenic CD4^+^ T cells were solubilized with lysis buffer with 1% SDS. The same amount of proteins (10 μg for each) were subjected to 10% SDS-PAGE (Ohya et al., 2014). The blots were incubated with an anti-K_2P_5.1 antibody (G-14 or H-170, Santa Cruz Biotechnology, Santa Cruz, CA, USA), and then incubated with anti-rabbit or anti-goat horseradish peroxidase-conjugated IgG (Millipore). An enhanced chemiluminescence detection system (GE Healthcare, Buckinghamshire, England) was used to detect the bound antibody. The resulting images were analyzed by a LAS-3000mini device (Fujifilm, Tokyo, Japan). The blots were also probed with an anti-β-actin (ACTB) antibody (Medical & Biological Laboratories, Nagoya, Japan).

### Measurement of the membrane potential and intracellular Ca^2+^ concentrations by fluorescent indicators

The membrane potential was measured using the fluorescent voltage-sensitive dye DiBAC_4_(3), as previously reported (Ohya et al., 2013, 2014). Glass bottom tissue culture dishes were coated with fibronectin (Wako Pure Chemical Industries, Osaka, Japan) in Ca^2+^ and Mg^2+^-free PBS at 50 μg/mL for 1 μg/cm^2^ at 4°C overnight. Cells were seeded onto fibronectin-coated dishes and cultured for 2 h at 37°C in 5% CO_2_ humidified incubator. Prior to the fluorescence measurements with DiBAC_4_(3), cells were incubated in normal HEPES buffer containing 100 nM DiBAC_4_(3) for 20 min at room temperature. The cells were continuously incubated with 100 nM DiBAC_4_(3) throughout the experiments. In membrane potential imaging, cells loaded with DiBAC_4_(3) were illuminated at a wavelength of 490 nm, and fluorescence images were recorded on the ORCA-Flash2.8 digital camera (Hamamatsu Photonics, Hamamatsu, Japan). Additionally, intracellular Ca^2+^ concentrations were measured using the fluorescent Ca^2+^ indicator dye Fura 2-AM. Cells were incubated with 10 μM Fura 2-AM in normal HEPES solution for 30 min at room temperature. Cells loaded with Fura 2-AM were alternatively illuminated at wavelengths of 340 and 380 nm, and fluorescence images were recorded. The fluorescent intensity of Fura 2 was expressed as measured 340/380 nm fluorescence ratios after background subtraction. Data collection and analyses were performed using an HCImage system (Hamamatsu Photonics). Images were measured every 5 s, and the values of fluorescent intensity (F) were determined by measuring the average for 1 min (12 images).

#### Flow cytometric analysis

Cell surface markers were analyzed with BD FACSCalibur (BD Biosciences, San Jose, CA, USA), which acquired at least 10,000 events, and gated according to forward- and side-scatter (Ohya et al., 2013). Data were analyzed using CellQuest software (BD Biosciences). The lymphocyte gate was established by analyzing the forward angle vs. right angle light scatter. The percentage of positive-staining cells was determined by comparing the test histograms with those obtained using a fluorescein isothiocyanate (FITC) or phycoerythrin (PE)-conjugated anti-CD4 antibody (FITC/PE-CD4), FITC-conjugated CD3 (FITC-CD3), PE-conjugated CD8 (PE-CD8), and PE-conjugated CD25 (PE-CD25) (BD Biosciences). After being incubated with the antibodies for 1 h at room temperature, excess antibodies were removed by repeated washing with PBS.

### Chemicals

The sources of pharmacological agents were as follows: clofilium tosylate (Sigma-Aldrich, Tokyo, Japan), DiBAC_4_(3), (Dojindo, Kumamoto, Japan), Fura 2-AM (Invitrogen), and 2-APB (Tocris Bioscience, Ellisville, MO, USA). All others were obtained from Sigma-Aldrich or Wako Pure Chemical Industries.

### Statistical analysis

Significant differences between two and among multiple groups were evaluated using the Student's *t*- and Welch's *t*-tests or Tukey's test after the *F*-test or ANOVA. Data that were non-normal distributed were analyzed using the Mann-Whitney *U*-test. Significance at *P* < 0.05 and *P* < 0.01 was indicated in the figures. Data are presented as means ± SE.

## Results

### Upregulated expression of the K_2P_5.1 K^+^ channel in splenic CD4^+^ T cells from the mouse model of DSS-induced inflammatory bowel disease

**Figure 1:**
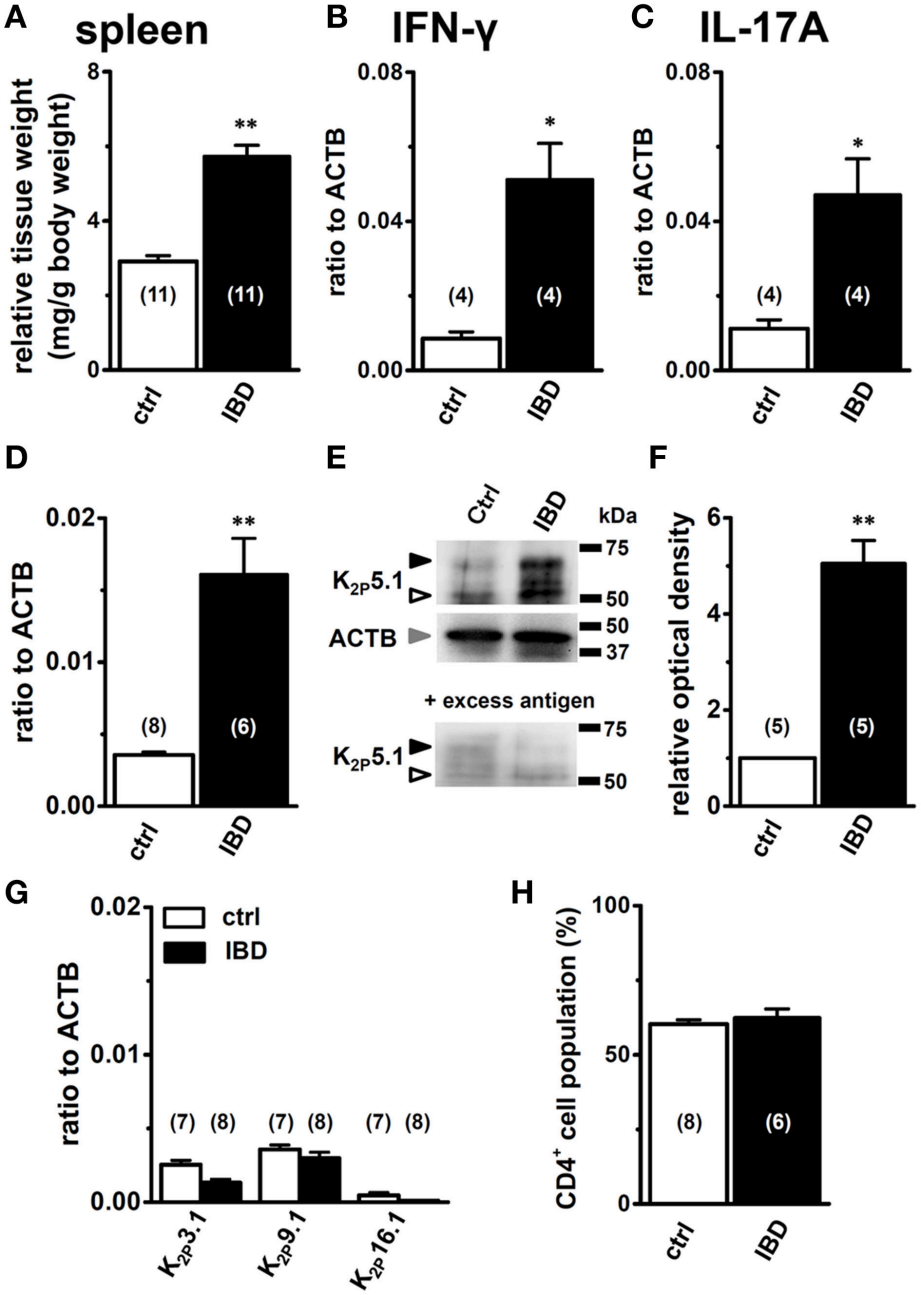
**Expression levels of K_2P_5.1 and pro-inflammatory cytokines in splenic CD4^+^ T lymphocytes of chemically-induced IBD model mice**. **(A)** spleen weight relative to body weight in control and IBD mice. **(B–D)** quantitative, real-time PCR assay for the expression of IFN-γ **(B)**, IL-17A **(C)**, and K_2P_5.1 **(D)** transcripts in the splenic CD4^+^ cells of control and IBD model mice. Expression levels are expressed as a ratio to β-actin (ACTB). **(E)** splenic CD4^+^ cell lysates were probed by immunoblotting with an anti-K_2P_5.1 (G-14) antibody. Molecular weight standards are shown in kilodaltons (kDa) on the right. Arrowheads indicate the migrating positions of K_2P_5.1 and ACTB proteins (open: K_2P_5.1, closed: glycosylated K_2P_5.1, gray: ACTB). Lower panel shows signals after the pre-incubation of a primary antibody with excess antigens. **(F)** summarized data obtained from **(E)** as the optical density of the band signal for K_2P_5.1 (open arrowhead) in the IBD model relative to that in normal mice. **(G)** Expression of alkaline pH-activated K_2P_ K^+^ channel subtype (K_2P_3.1, 9.1, and 16.1) transcripts in the splenic CD4^+^ T cells of control and IBD model mice. **(H)** the percentages of the CD4^+^ T cell population relative to CD3+ T cells were analyzed by flow cytometry, acquiring at least 10,000 events. Results were expressed as means ± SEM. Numbers used for the experiments are shown in parentheses. ^*^, ^**^P < 0.05, 0.01 vs. control mice (ctrl).

Mice were sacrificed 7 days after the administration of 5.0% (wt/vol) DSS and tissue samples were collected. Control mice were given drinking water only. As previously reported (Ohya et al., 2014), severe macroscopic symptoms (colitis and fecal blood) were detected in DSS-induced IBD model mice, and the scores of colonic inflammation and crypt damage were significantly higher in IBD model mice than in control mice (not shown). As reported by Pereira et al. (1987), enlargement of the spleen, “splenomegaly,” was detected in IBD model mice (Figure 1A), and a significant increase in the transcriptional expression of the Th1 cytokine interferon-γ (IFN-γ) and Th17 cytokine interleukin-17 (IL-17) was detected (Figures 1B,C), without any changes in the Th2 cytokine IL-4 (not shown). The flow cytometric analysis showed no significant differences of the CD4^+^ phenotypic ratio between isolated splenic CD3^+^ T cells from both groups (Figure 1H).

The quantitative, real-time PCR assay showed that the expression level of the K_2P_5.1 transcripts was significantly higher in the splenic CD4^+^ T cells of IBD model mice (IBD) than in control mice (ctrl) (Figure 1D). The expression levels of K_2P_5.1 relative to ACTB (in arbitrary units) were 0.0036 ± 0.0002 and 0.0161 ± 0.0025 in the splenic CD4^+^ T cells of control mice (*n* = 8) and IBD model mice (*n* = 6, *P* < 0.01), respectively. The western blot examination showed that the amount of the K_2P_5.1 protein (~50 kDa) in the cell lysate of splenic CD4^+^ T cells was higher in IBD model mice than in control mice (Figure 1E, upper panel, open arrowhead), which was consistent with the results obtained by real-time PCR examination. The amount of the glycosylated K_2P_5.1 protein (~65 kDa, Figure 1E, upper panel, closed arrowhead) was also higher in the IBD model. The optical density for the K_2P_5.1 protein band signal (~50 kDa) relative to that for ACTB (Figure 1E, middle panel) was calculated using Image J software (Ver. 1.42, NIH), and its protein expression level in control mice was expressed as 1.00. The relative optical density in the IBD model was 5.05 ± 0.48 (*n* = 5 for each, *P* < 0.01) (Figure 1F). The two band signals specific for an anti-K_2P_5.1 antibody (G-14) disappeared with a pre-incubation with excess antigens (Figure 1E, lower panel). Similar results were obtained when another anti-K_2P_5.1 antibody (H-170) was used as the primary antibody (Supplementary Figure 1). In addition, the expression levels of the other alkaline pH-activated K_2P_ channel subtypes, K_2P_3.1/TASK1, K_2P_9.1/TASK3, and K_2P_16.1/TALK1 transcripts, were less abundant in the IBD model: < 0.004 in arbitrary units (Figure 1G).

### Enhancement in alkaline pH_e_-induced hyperpolarizing responses in splenic CD4^+^ T cells of IBD model mice

**Figure 2:**
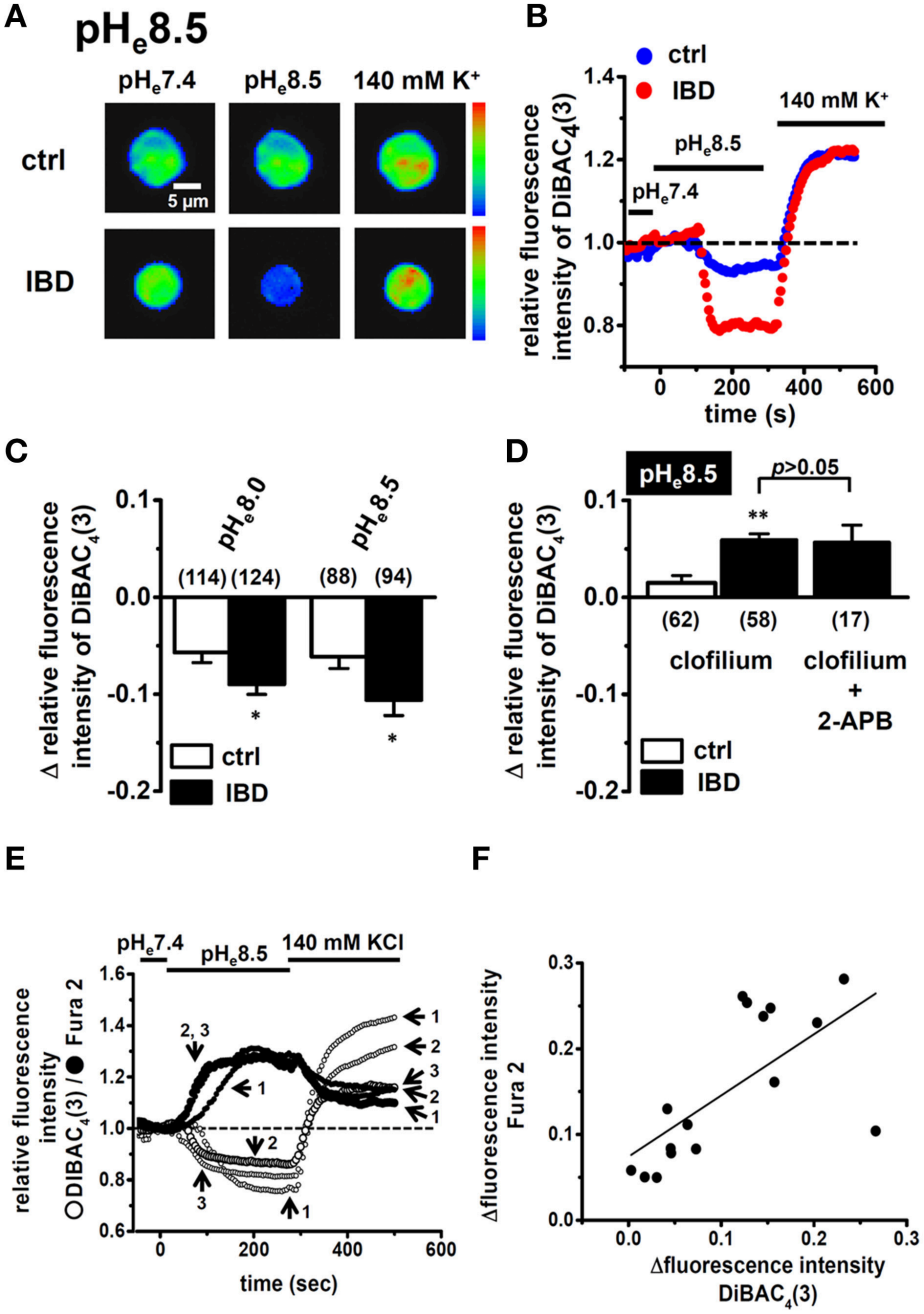
**Effects of alkaline pH_e_ on membrane potential and intracellular Ca^2+^ mobilization in splenic CD4^+^ T lymphocytes of IBD model mice**. **(A,B)** measurement of alkaline pH_e_ (pH_e_ 8.5)-induced hyperpolarizing responses in splenic CD4^+^ T cells using the voltage-sensitive fluorescent dye DiBAC_4_(3). Images **(A)** are shown in pseudo-colors from blue (low intensity) to red (high intensity) in the bar. **(B)** shows the time course of changes in the fluorescence intensity of DiBAC_4_(3). The fluorescence intensity of DiBAC_4_(3) at 0 s (pH_e_ 7.4) is expressed as 1.0. **(C)** summarized data are shown as alkaline pH_e_ (pH_e_ 8.0 and 8.5)-induced fluorescence changes [Δrelative fluorescence intensity of DiBAC_4_(3)] in splenic CD4^+^ T cells of control and IBD model mice. **(D)** effects of clofilium (5 μM) and the combination of clofilium (5 μM) and 2-APB (10 μM) on alkaline-pH_e_ (pH_e_ 8.5)-induced hyperpolarizing responses in the splenic CD4^+^ T cells of control and IBD model mice. Cells were pre-treated with clofilium and/or 2-APB for 30 min. **(E,F)** simultaneous measurements of changes in the membrane potential and intracellular Ca^2+^ concentrations by hyperpolarizing (pH_e_ 8.5) and depolarizing (140 mM KCl) stimulations using DiBAC_4_(3) and Fura 2, a Ca^2+^ indicator. **(E)** shows the time course of changes in their fluorescence intensities, and summarizing data are shown in **(F)**. Results were expressed as means ± SEM. Numbers used for the experiments are shown in parentheses. ^*^, ^**^P < 0.05, 0.01 vs. control mice (ctrl).

To perform functional analysis of K_2P_5.1 in splenic CD4^+^ T cells, we measured the hyperpolarizing responses induced by the alkalization of extracellular pH (pH_e_) (pH_e_8.0 and 8.5) using the membrane potential-sensitive dye, DiBAC_4_(3). To confirm living cells, 140 mM high K^+^-induced depolarization was measured at the end of protocol. As shown in Figure 2A, the fluorescence intensity of DiBAC_4_(3) decreased with the application of alkaline pH_e_ (pH_e_8.5). The peak amplitude of the relative fluorescence intensity of DiBAC_4_(3) was lower in IBD model mice than in control mice (Figure 2B). When alkaline pH_e_-induced hyperpolarizing responses (pH_e_8.0 and 8.5) were expressed as changes in the relative fluorescence intensity of DiBAC_4_(3) [Δ relative fluorescence intensity of DiBAC_4_(3)], they were significantly larger in IBD model mice than in control mice (Figure 2C). Under alkaline pH_e_ conditions, high K^+^-induced increases in the fluorescence intensity of DiBAC_4_(3) were almost the same as those under normal pH_e_ (pH_e_7.4) conditions (Figure 2B). In the presence of the non-specific K_2P_5.1 blocker, clofilium (5 μM), alkaline pH_e_ (pH_e_8.5)-induced hyperpolarizing responses disappeared in both groups. Significantly larger depolarizing responses were observed in IBD model mice (Figure 2D), suggesting that alkaline pH_e_-induced hyperpolarizing responses via the activation of K_2P_5.1 in IBD model mice were underestimated. Clofilium also inhibits voltage-gated K^+^ channel K_*V*_1.3, one of main K^+^ channels in T cells, however, 4-aminopyridine (5 mM), a K_*V*_1.3 blocker, did not induce any significant differences in the alkaline pH_e_ (pH_e_ 8.5)-induced hyperpolarizing responses [Δ relative fluorescence intensity of DiBAC_4_(3) = −0.17±0.04 (*n* = 19)]. A recent study demonstrated that the Ca^2+^-release activated Ca^2+^ (CRAC) channel, Orai1, which contributes to the store-operated entry of Ca^2+^ into T cells, was activated by alkaline pH (Beck et al., 2014), suggesting that the function of Orai1 was upregulated in the splenic CD4^+^ T cells of IBD model mice. However, depolarizing responses were not suppressed by the pre-treatment with the Orai1 blocker 2-APB (10 μM) for more than 5 min (*P* < 0.01) (Figure 2D), and the expression levels of Orai1 transcripts were also not changed between the two groups (not shown). We subsequently observed a relationship between alkaline pH_e_-induced hyperpolarization responses and [Ca^2+^]_i_ rises via CRAC channels in the splenic CD4^+^ T cells of IBD model mice by simultaneously measuring DiBAC_4_(3) and Fura 2 signals (Figure 2E). As shown in Figure 2F, alkaline pH_e_-induced hyperpolarization responses positively correlated with [Ca^2+^]_*i*_ rises in the splenic CD4^+^ T cells of IBD model mice (*R*^2^ = 0.41). The alkaline pH_e_-induced [Ca^2+^]_*i*_ rises were suppressed by about 40% by the pre-treatment with 2-APB (10 μM) (not shown).

### Higher expression level of K_2P_5.1 in the inflammatory CD4^+^CD25^−^ subset than in the regulatory CD4^+^CD25^+^ subset

**Figure 3:**
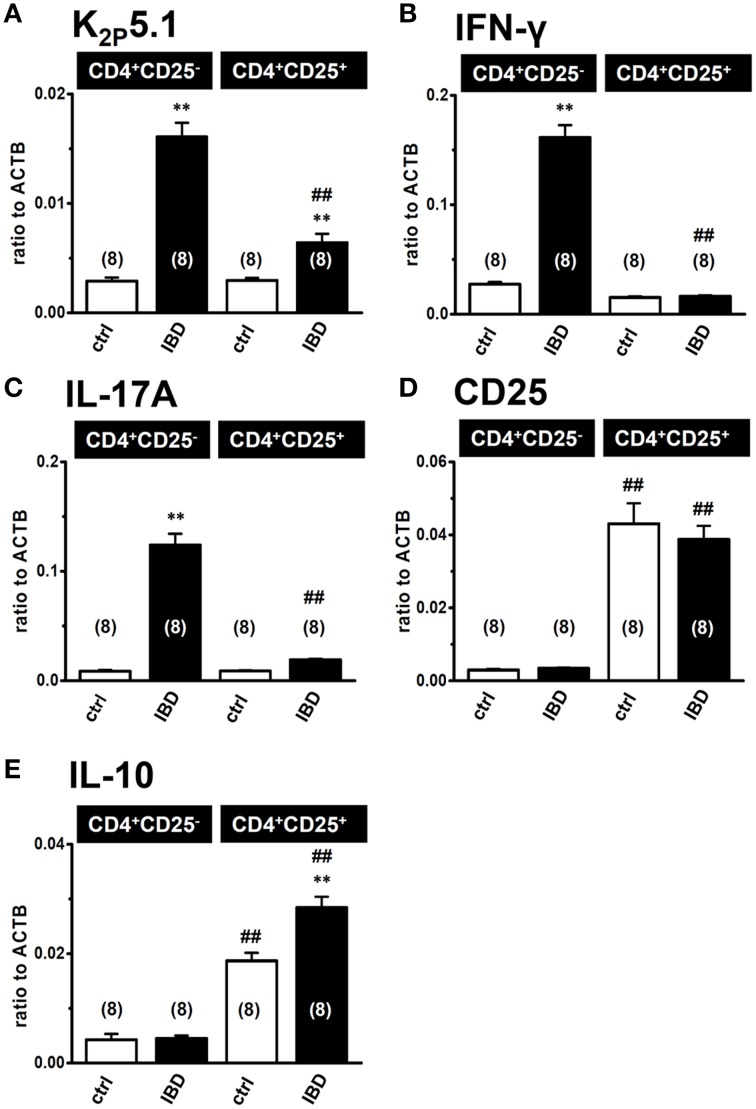
**Expression levels of K_2P_5.1 and pro- and anti-inflammatory cytokine transcripts in splenic CD4^+^CD25^−^ and CD4^+^CD25^+^ subsets of IBD model mice. (A–E)** real-time PCR assay for the expression of K_2P_5.1 **(A)**, IFN-γ **(B)**, IL-17A **(C)**, CD25 **(D)**, and IL-10 **(E)** transcripts in the splenic CD4^+^CD25^−^ and CD4^+^CD25^+^ T cells of control and IBD model mice. Results were expressed as means ± SEM. Numbers used for the experiments are shown in parentheses. ^**^P < 0.01 vs. control mice (ctrl), ^##^*P* < 0.01 vs. the CD4^+^CD25^−^ subset.

When K_2P_5.1 is upregulated in Th1/Th17 cells producing IFN-γ/IL-17, the inhibition of K_2P_5.1 may suppress the disease progression of IBD. However, when K_2P_5.1 is upregulated in regulatory T cells producing IL-10, the inhibition of K_2P_5.1 may promote the disease progression of IBD. In order to clarify the pathophysiological significance of the K_2P_5.1 K^+^ channel in IBD, we examined differences in the expression levels of K_2P_5.1 between the splenic CD4^+^CD25^−^ and CD4^+^CD25^+^ subsets in IBD model mice using a real-time PCR assay. As shown in Figure 3, the proinflammatory Th1/Th17 cytokines IFN-γ (Figure 3B) and IL-17A (Figure 3C) transcripts were expressed at high levels in the CD4^+^CD25^−^ subset of IBD model mice, whereas the natural regulatory T cell (T_reg_) markers CD25 (Figure 3D), IL-10 (Figure 3E), and Foxp3 (not shown) were predominantly expressed in the CD4^+^CD25^+^ subset. As shown in Figure 3A, K_2P_5.1 transcripts were expressed at significantly higher levels in the CD4^+^CD25^−^ subset in IBD model mice than in control mice. Significant increases in the expression levels of the K_2P_5.1 transcripts were also found in the CD4^+^CD25^+^ subset of IBD model mice (*P* < 0.01 vs. control) (Figure 3A); however, these increases were significantly lower than those observed in the CD4^+^CD25^−^ subset of IBD model mice (*P* < 0.01). Correspondingly, similar levels of alkaline pH_e_-induced hyperpolarizing responses (pH_e_8.5) were observed in the CD4^+^CD25^−^ subset of IBD model mice but not in the CD4^+^CD25^+^ subset (not shown). These results suggested that the pharmacological inhibition of K_2P_5.1 is an effective strategy for the treatment of IBD.

### Decreased susceptibility to the pathogenesis of IBD in K_2P_5.1 knockout mice

**Figure 4:**
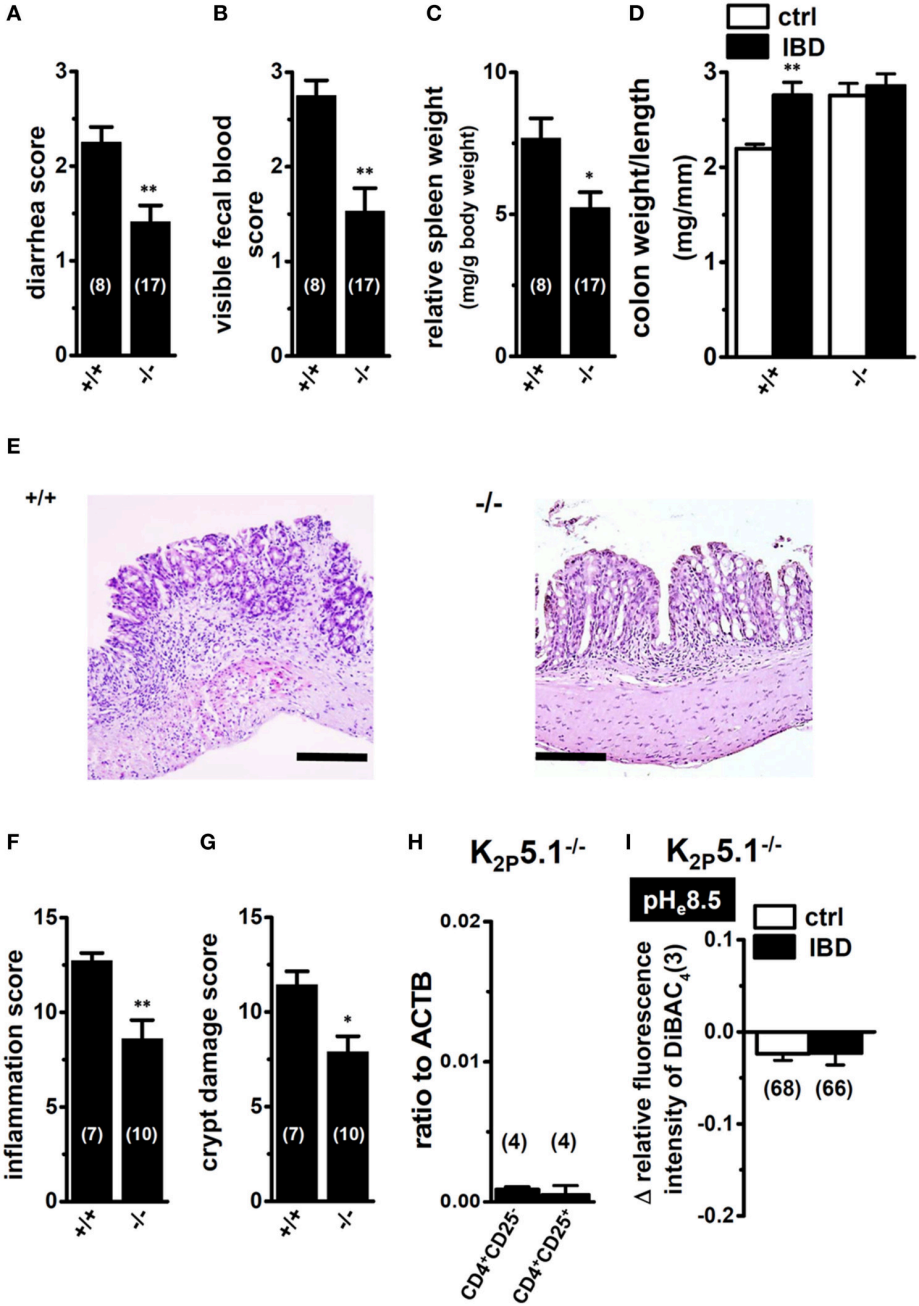
**Macroscopic disease activities and histopathological assessments in the IBD model from wild-type (+/+) and homozygous K_2P_5.1 knockout (-/-) mice. (A,B)** Seven days after DSS exposure, diarrhea, and visible fecal blood severities were scored on a 0–3 scale (0, Normal pellets; 1, Slightly loose feces; 2, Loose feces; 3, Watery diarrhea) and a 0–3 scale (0, Normal; 1, Slightly bloody; 2, Bloody; 3, Blood in the whole colon), respectively, in K_2P_5.1^+/+^ and K_2P_5.1^−/−^ mice. **(C)** spleen weights relative to body weights in K_2P_5.1^+/+^ and K_2P_5.1^−/−^ mice. **(D)** the colon weight/length ratio was measured in K_2P_5.1^+/+^ and K_2P_5.1^−/−^ mice. **(E–G)** Photographs of colon sections from K_2P_5.1^+/+^ and K_2P_5.1^−/−^ mice with H&E staining (magnification, X100) **(E)**. Scale bar, 100 μm. Colonic inflammation **(F)** and crypt damage **(G)** were assessed histologically. **(H)** Expression of K_2P_5.1 transcripts relative to ACTB transcripts in CD4^+^CD25^−^ and CD4^+^CD25^+^ subsets from K_2P_5.1^−/−^ mice. **(I)** summarized data are shown as alkaline pH_e_ (pH_e_ 8.5)-induced hyperpolarizing responses in the splenic CD4^+^ T cells of control and IBD model mice. Results were expressed as means ± SEM. The numbers used for the experiments are shown in parentheses. ^*^, ^**^P < 0.05, 0.01 vs. K_2P_5.1^+/+^ **(D)**.

As described above, the lack of selective pharmacological agents represents the main difficulty in carrying out experimental studies on K_2P_5.1. Therefore, we examined the effects of the genetic inhibition of K_2P_5.1 on inflammatory responses during acute IBD in K_2P_5.1 homozygous knockout (K_2P_5.1^−/−^) mice. As reported in our recent study (Ohya et al., 2014), severe clinical symptoms and enlargement of the spleen were observed in wild-type K_2P_5.1^+/+^ mice (Figures 4A–C, left columns). A significant protective effect against IBD was observed in K_2P_5.1^−/−^ mice (Figures 4A,B, right columns). The diarrhea scores were 2.25 ± 0.16 (*n* = 8) and 1.41 ± 0.17 (*n* = 17) in K_2P_5.1^+/+^ and K_2P_5.1^−/−^ mice, respectively (*P* < 0.01 vs. K_2P_5.1^+/+^). The degrees of splenomegaly was also significantly lower in K_2P_5.1^−/−^ mice (*P* < 0.05 vs. K_2P_5.1^+/+^). Furthermore, colonic inflammation and crypt damage were quantified by colon weight/length ratio measurements and histological scoring. As shown in Figure 4D, colonic wall thickening was significantly increased in K_2P_5.1^+/+^ mice, whereas no significant differences between control and IBD were observed in K_2P_5.1^−/−^ mice. Histological assessments of colonic inflammation and crypt damage also revealed that both scores were significantly lower in K_2P_5.1^−/−^ mice than in K_2P_5.1^+/+^ mice (*P* < 0.01 for the inflammation score, *P* < 0.05 for the crypt damage score) (Figures 4E–G). No significant differences in the degrees of colitis, colonic inflammation, or crypt damage were observed in heterozygous knockout mice (K_2P_5.1^+/−^) [diarrhea score: 1.95 ± 0.20 (*n* = 22), visible fecal blood score: 2.32 ± 0.17 (*n* = 22), inflammation score: 10.43 ± 0.87 (*n* = 7), crypt damage score: 9.86 ± 0.86 (*n* = 7)]. In K_2P_5.1^−/−^ and K_2P_5.1^+/−^ mice drinking water only for 7 days, all scores assessed in this study were 0 (not shown). In addition, in the splenic CD4^+^CD25^−^ and CD4^+^CD25^+^ cells of K_2P_5.1^−/−^ mice, the expression level of K_2P_5.1 transcripts almost disappeared (Figure 4H), and alkaline pH_e_ (pH_e_8.5)-induced hyperpolarizing responses were also very small in the splenic CD4^+^ T cells of K_2P_5.1^−/−^ mice (Figure 4I). These results suggest that K_2P_5.1 inhibitors may be efficacious in patients with IBD.

## Discussion

The activation of K^+^ channels in T cells promotes Ca^2+^ influx, thereby indirectly modulating Ca^2+^ signaling (Vig and Kinet, 2009). Two K^+^ channel subtypes, the voltage-gated K_*V*_1.3 and intermediate-conductance Ca^2+^-activated K^+^ channel K_Ca_3.1, are known to mainly function in T and B lymphocytes (Di Sabatino et al., 2009). The physiological role of the two-pore domain K^+^ channel K_2P_5.1 was recently clarified in lymphocytes (Nam et al., 2011; Cid et al., 2013; Shin et al., 2014), and several studies have shown the pathophysiological impact of the upregulation of the two-pore domain K^+^ channel K_2P_5.1 in CD4^+^ T cells on the pathogenesis of autoimmune diseases such as rheumatoid arthritis and multiple sclerosis (Bittner et al., 2010a, 2011). However, the pathophysiological impact of K_2P_5.1 in diseases associated with Th1 and Th17 cytokine profiles has not yet been examined using *in vivo* animal models. This is the first study to employ chemically-induced IBD model mice to characterize the pathophysiological role of K_2P_5.1 in mature CD4^+^ T cells. The main results of the present study were as follows: (1) Facilitation of the expression level and functional activity of the K_2P_5.1 K^+^ channel in splenic CD4^+^ T cells of IBD model mice, especially in the CD4^+^CD25^−^ subset (Figures 1, 2), (2) Decreased disease activity index (diarrhea, bloody feces, and weight loss) and histopathological scores (colonic inflammation and crypt damage) in homozygous K_2P_5.1-deficient (K_2P_5.1^−/−^) mice (Figure 4). We were unable to elucidate the mechanism underlying the transcription regulation of K_2P_5.1 in the splenic CD4^+^ T cells of IBD model mice. The voltage-gated K^+^ channel K_*V*_1.3 and Ca^2+^-activated K^+^ channel K_Ca_3.1 are the main K^+^ conductance channels in CD4^+^ T cells. Although both transcripts were expressed in splenic CD4^+^CD25^−^ cells at high levels, no significant differences were observed in their expression levels between control and IBD model mice (not shown). These results suggested that the upregulation of K_2P_5.1 played an important role in the changes observed in T cell Ca^2+^ signaling in IBD pathogenesis, and may affect the facilitation of proliferation and infiltration of splenic CD4^+^ T cells. K_2P_5.1 also contributes to the regulation of osmotic volume, regulatory volume decreases (RVD), in several types of cells including T cells (Niemeyer et al., 2001; Bobak et al., 2011; Andronic et al., 2013), which participate in cell cycle progression. Therefore, a dysfunction in cellular volume homeostasis via the upregulation of K_2P_5.1 may also be involved in dysregulated cellular functions in the CD4^+^ T cells of IBD model mice.

**Figure 5:**
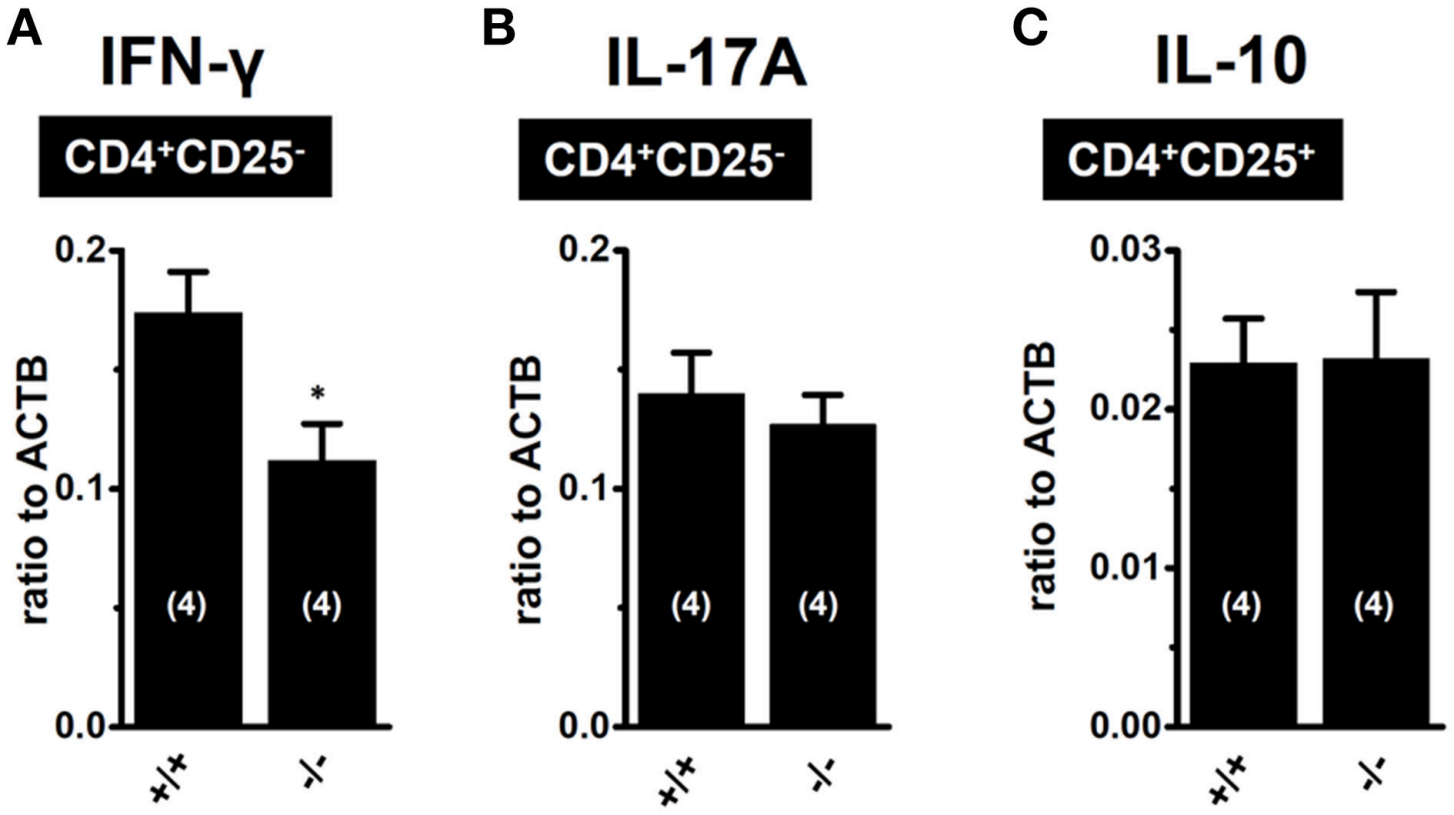
**Expression levels of IFN-γ and IL-17A transcripts in CD4^+^CD25^−^ subset of IBD models from K_2P_5.1^+/+^ (+/+) and K_2P_5.1^−/−^ (-/-) mice (A,B), and IL-10 transcripts CD4^+^CD25^+^ subset of IBD models from K_2P_5.1^+/+^ (+/+) and K_2P_5.1^−/−^ mice (-/-) (C)**. Expression levels were measured using a real-time PCR assay. Results were expressed as means ± SE. Numbers used for the experiments are shown in parentheses. ^*^P < 0.01 vs. K_2P_5.1^+/+^ mice.

As shown in Figure 3A, the upregulation of K_2P_5.1 was greater in splenic CD4^+^CD25^−^ T cells of IBD model mice than in CD4^+^CD25^+^ T cells. CD4^+^CD25^−^ T cells include pro-inflammatory Th1 and Th17 subsets, and an increase was observed in the transcription of IFN-γ and IL-17A in the splenic CD4^+^CD25^−^ T cells of IBD model mice (Figures 3B,C). Due to the lack of a selective K_2P_5.1 inhibitor, we were unable to examine the effects of its *in vivo* administration on the disease activity index and histopathological scores in IBD model mice in order to clarify the pathophysiological significance of K_2P_5.1 in IBD. However, we showed that the genetic knockdown of K_2P_5.1 significantly protected against IBD via the amelioration of severe colitis and colonic inflammation using homozygous K_2P_5.1 knockdown K_2P_5.1^−/−^ mice (Figure 4). Furthermore, the expression levels of the IFN-γ transcripts from the pro-inflammatory Th1 subset were significantly lower in the CD4^+^CD25^−^ cells of the IBD model from K_2P_5.1^−/−^ mice than from K_2P_5.1^+/+^ mice (Figure 5A), and no significant differences were noted in the expression levels of IL-17 in CD4^+^CD25^−^ cells and IL-10 in CD4^+^CD25^+^ cells between IBD models from K_2P_5.1^+/+^ and K_2P_5.1^−/−^ mice (Figures 5B,C). Similar findings were obtained with the genetic and pharmacological knockdown of the Ca^2+^-activated K^+^ channel K_Ca_3.1 in an IBD model (Di et al., 2010; Ohya et al., 2014; Ohya and Imaizumi, 2014). It has been reported that the function of Th1 was impaired in an IBD model from K_Ca_3.1^−/−^ mice, whereas that of Th17 was normal (Di et al., 2010). We recently demonstrated the inhibition of IFN-γ transcription, but not that of IL-17A by the *in vivo* administration of selective K_Ca_3.1 inhibitors in a DSS-induced IBD model (Ohya et al., 2014). Therefore, the development of small molecule K_2P_5.1 modulators may represent therapeutic and/or preventative strategies for the treatment of inflammatory and autoimmune disorders and cancer, and the establishment of a novel high-throughput screening system to detect these modulators currently required (Bagriantsev et al., 2013). Furthermore, novel findings on the regulatory molecules of K_2P_5.1 will provide insights into the inhibition of K_2P_5.1.

Regulatory T (T_reg_) cells play an important role in the maintenance of intestinal homeostasis, and T_reg_ cells are considered to be beneficial for IBD therapy (Boden and Snapper, 2008; Himmel et al., 2012; Gibson et al., 2013; Mayne and Williams, 2013). As shown in Figure 3A, the expression levels of K_2P_5.1 transcripts of K_2P_5.1 were significantly increased in the splenic CD4^+^CD25^+^ T cells of IBD model mice. This subset also included Foxp3-positive cells (not shown) and, thus, is referred to as naturally-occurring regulatory T cells. In B lymphocytes, the production of the anti-inflammatory cytokine IL-10 was shown to be stimulated by Ca^2+^ influx via the CRAC channel (Matsumoto et al., 2011; Baba et al., 2014). Therefore, the upregulation of K_2P_5.1 may strengthen the production of IL-10 in the natural T_reg_ cells of IBD model mice, and the activation of K_2P_5.1 may improve the pathogenesis of IBD. In the present study, the expression of IL-10 was significantly higher in the natural T_reg_ cells of IBD model mice than in those of control mice (Figure 3E). No significant changes in the expression of IL-10 were found in natural T_reg_ cells of the IBD model from K_2P_5.1^−/−^ mice (Figure 5C); however, the long-lasting dysregulation of Ca^2+^ signaling elicited by a K_2P_5.1 deficiency may be compensated by the upregulation of other K^+^ channel(s), and, thus, the expression of IL-10 may be maintained within normal ranges in the T_reg_ cells of the IBD model from K_2P_5.1^−/−^ mice. Further studies are needed in order to clarify the pathophysiological role of K_2P_5.1 in the natural T_reg_ cells of IBD.

In conclusion, the results of the present study suggested that the background K^+^ channel K_2P_5.1 in splenic CD4^+^ T cells was involved in the pathogenesis of IBD using a chemically-induced IBD model and provided evidence for the K_2P_5.1 K^+^ channel as a potential therapeutic target for suppressing the progression of IBD. Dysregulated K_2P_5.1 may stimulate the Th1 imbalance in the process of intestinal inflammation. The lack of selective K_2P_5.1 blockers may facilitate future research on a novel high-throughput screening system to detect small molecule K_2P_5.1 modulators and the regulatory mechanisms underlying K_2P_5.1 transcription, translation, and protein degradation in IBD.

## Author contributions

SN, HK, and SO participated in research design. SN, MM, HK, MF, and SO conducted molecular biological and biochemical experiments and performed data analyses. SN, MM, AS, MI, and HK conducted fluorescence imaging experiments and performed data analyses. SN, HN, SM, MM conducted H&E staining experiments and performed data analyses. SN, AS, HK, EK, SM, MM, NK, MA, KA, and SO contribute to the maintenance of knockout mice and preparation of IBD model mice. SN, HK, and SO contributed to the writing of the manuscript.

## Funding

This work was supported by a Grant-in Aid for Scientific Research (C) (No. 25460111) by The Japan Society for the Promotion of Science (JSPS), a grant for the Supported Program for the Strategic Research Foundation at Private Universities, 2013-2017 (S1311035) from the Ministry of Education, Culture, Sports, Science and Technology (MEXT), the Mochida Memorial Foundation for Medical and Pharmaceutical Research, and the Uehara Memorial Foundation (to SO), a research grant from The Promotion and Mutual Aid Cooperation for Private Schools of Japan (Kyoto Pharmaceutical University and Aichi-Gakuin University).

### Conflict of interest statement

The authors declare that the research was conducted in the absence of any commercial or financial relationships that could be construed as a potential conflict of interest.

## Abbreviations

K_2P_: two-pore domain K^+^ (channel)
TASK: TWIK-related acid-sensitive K^+^ (channel)
TWIK: tandem of P-domains in a weakly inward rectifying K^+^ (channel)
TALK: TWIK-related alkaline pH-activated K^+^ (channel)
IBD: inflammatory bowel disease
DSS: dextran sodium sulfate
HEPES: 4-(2-hydroxyethyl)-1-piperazineethanesulfonic acid
DiBAC_4_(3): bis-(1,3-dibutylbarbituric acid)trimethine oxonol
Fura 2-AM: acetoxymethyl 2-[5-[bis[(acetoxymethoxy-oxo-methyl)methyl]amino]-4-[2-[2-[bis[(acetoxymethoxy-oxo-methyl)methyl]amino]-5-methyl-phenoxy]ethoxy]benzofuran-2-yl]oxazole-5-carboxylate
FCM: flow cytometry
IFN: interferon
IL: interleukin
pH_e_: extracellular pH
CD: cluster of differentiation or Crohn's disease
UC: ulcerative colitis
FITC: fluorescein isothiocyanate
PE: Phycoerythrin
2-APB: 2-aminoethoxydiphenyl borate
Orai: Ca^2+^ release-activated Ca^2+^ (channel)
STIM: stromal interaction molecule
TRPV: transient receptor potential, vanilloid
TRPA: transient receptor potential, subfamily A
K_Ca_: Ca^2+^-activated K^+^ (channel)
K_*V*_: voltage-gated K^+^ (channel).
