# Supplementary material for: Pathophysiological significance of the two-pore domain K+ channel K2P5.1 in splenic CD4+CD25− T cell subset from a chemically-induced murine inflammatory bowel disease model
Source: Front Physiol. 2015 Oct 27;6:299. doi: 10.3389/fphys.2015.00299 (PMC4621418; doi:10.3389/fphys.2015.00299)
Supplement: Supplementary file 1 [file DataSheet1.DOCX]

***Supplementary Material***

**Pathological significance of the two-pore domain K^+^ channel K_2P_5.1 in splenic CD4^+^ T cells from a chemically-induced murine inflammatory bowel disease model**

**Sawa Nakakura, Miki Matsui, Aya Sato, Mizuki Ishii, Kyoko Endo, Sayaka Muragishi, Miki Murase, Natsumi Kurokawa, Hiroaki Kito, Masanori Fujii, Masatake Araki, Kimi Araki, Susumu Ohya***

*** Correspondence:** Susumu Ohya: [sohya@mb.kyoto-phu.ac.jp](mailto:sohya@mb.kyoto-phu.ac.jp)

**Supplementary Data**

Supplementary Material should be uploaded separately on submission. Please include any supplementary data, figures and/or tables.

Supplementary material is not typeset so please ensure that all information is clearly presented, the appropriate caption is included in the file and not in the manuscript, and that the style conforms to the rest of the article.

1. **Supplementary Figures and Tables**

## Supplementary Figures

Supplementary Figure 1. Expression level of K_2P_5.1 proteins in splenic CD4^+^ T lymphocytes of chemically-induced IBD model mice. A: splenic CD4^+^ cell lysates were probed by immunoblotting with an anti-K_2P_5.1 (H-170) antibody. Molecular weight standards are shown in kilodaltons (kDa) on the right. Arrowheads indicate the migrating positions of K_2P_5.1 and ACTB proteins. B: summarized data obtained from ‘A’ as the optical density of the band signal for K_2P_5.1 in the IBD model relative to that in normal mice. Results were expressed as means ± SEM. Numbers used for the experiments are shown in parentheses. ***P*<0.01 vs. control mice.
